# LSD-assisted therapy in patients with anxiety: open-label prospective 12-month follow-up

**DOI:** 10.1192/bjp.2024.99

**Published:** 2024-09

**Authors:** Friederike Holze, Peter Gasser, Felix Müller, Manuel Strebel, Matthias E. Liechti

**Affiliations:** Clinical Pharmacology and Toxicology, Department of Biomedicine and Department of Clinical Research, University Hospital Basel and University of Basel, Switzerland; Department of Pharmaceutical Sciences, University of Basel, Switzerland; Clinic Dr Peter Gasser, Solothurn, Switzerland; Psychiatric University Hospital, University of Basel, Switzerland

**Keywords:** LSD, anxiety, RCT, psychedelics, depression

## Abstract

**Background:**

Anxiety disorders are a major public health burden with limited treatment options.

**Aims:**

We investigated the long-term safety and efficacy of lysergic acid diethylamide (LSD)-assisted therapy in patients with anxiety with or without life-threatening illness.

**Method:**

This study was an *a priori*-planned long-term follow-up of an investigator-initiated, two-centre trial that used a double-blind, placebo-controlled, two-period, random-order, crossover design with two sessions with either oral LSD (200 μg) or placebo per period. Participants (*n* = 39) were followed up 1 year after the end-of-study visit to assess symptoms of anxiety, depression and long-term effects of psychedelics using Spielberger's State-Trait Anxiety Inventory–Global (STAI-G), the Beck Depression Inventory (BDI), the Persisting Effects Questionnaire and measures of personality traits using the NEO**-**Five-Factor Inventory.

**Results:**

Participants reported a sustained reduction of STAI-G scores compared with baseline (least square means (95% CI) = −21.6 (−32.7, −10.4), *d* = 1.04, *P* < 0.001, for those who received LSD in the first period (94 weeks after the last LSD treatment) and −16.5 (−26.2, −6.8), *d* = 1.02, *P* < 0.05, for those who received LSD in the second period (68 weeks after the last LSD treatment)). Similar effects were observed for comorbid depression with change from baseline BDI scores of −8.1 (−13.2, −3.1), *d* = 0.71, *P* < 0.01, and −8.9 (−12.9, −4.9), *d* = 1.21, *P* < 0.01, for the LSD-first and placebo-first groups, respectively. Personality trait neuroticism decreased (*P* < 0.0001) and trait extraversion increased (*P* < 0.01) compared with study inclusion. Individuals attributed positive long-term effects to the psychedelic experience.

**Conclusions:**

Patients reported sustained long-term effects of LSD-assisted therapy for anxiety.

Anxiety disorders are a major public health burden. Treatment options are limited and may take several weeks to yield reductions of anxiety.^1^ Additionally, anxiety disorders often exhibit a chronic course, with many individuals facing recurring episodes.^2^ Therefore, novel treatment options are needed.

Emerging research shows that psychedelics, such as lysergic acid diethylamide (LSD) and psilocybin, might have promise for the treatment of psychiatric illnesses, such as anxiety disorders and depression.^3–7^ Several recent trials showed rapid and sustained symptom reductions of ratings of anxiety and depression after single-dose treatments.^4,8,9^ The primary analysis of the present crossover trial, including the primary end-point at 16 weeks post-LSD treatment compared with placebo, showed significant, rapid and sustained reductions of anxiety and comorbid depression in people with anxiety disorders with or without life-threatening illness (LTI) compared with placebo, and a further decrease in symptoms in the group who first received LSD after the crossover during treatment with placebo.^10^ It remains unclear, however, how long-lasting and sustained these symptom reductions are. A recent long-term follow-up study in individuals with major depressive disorder, for example, showed sustained reductions of depressive symptoms at the 12-month follow-up,^11^ and a study in people with cancer-related psychiatric distress showed anxiety and depression reductions at >3–4 years.^12^

Psychedelics may also have effects on personality traits.^13–15^ Two recent studies were conducted in participants with major depressive disorder who were treated with psilocybin. One study reported decreases in the personality domains neuroticism and introversion, and increases in openness.^16^ The other study reported decreases in neuroticism 6 months after treatment with psilocybin,^17^ but decreases in neuroticism were also present in their control group that was treated with escitalopram, indicating that changes in personality might be part of the therapeutic response in mood disorders.^18^ The personality traits neuroticism and extraversion have been linked to mood disorders. Specifically, high ratings in neuroticism and low ratings in extraversion were shown to be predictors of depression and anxiety.^19–21^

Although initial results for psychedelic-assisted therapy in people with anxiety disorders seem favourable, gaps remain with regard to long-term safety and efficacy. We aimed to investigate the long-term safety and efficacy of LSD-assisted therapy at 12-month follow-up in comparison with baseline measures using Spielberger's State-Trait Anxiety Inventory (STAI), the Beck Depression Inventory (BDI) and the Symptom-Check-List-90-R (SCL-90-R). We hypothesised effects would not be sustained up to the 12-month follow-up. We also explored long-term effects, such as changes in personality traits and positive life changes, that were attributed to the psychedelic experience.

## Method

### Study design and participants

The present study included an *a priori*-planned long-term (12-month) follow-up after the end-of-study visit of a double-blind, placebo-controlled, two-period, random-order, crossover study with two LSD (200 μg) sessions and two placebo sessions and five study visits per period in patients with anxiety disorders that were or were not associated with LTI. The order of administration was random and counterbalanced. For those who received LSD in the first period, the follow-up was assessed 94 weeks after the last LSD treatment. For those who received placebo in the first period, the follow-up was assessed 68 weeks after the last LSD treatment. The study was an investigator-initiated, two-centre trial, with one study centre at the University Hospital Basel, Switzerland, and the other study centre at the Clinic Dr Peter Gasser**,** Solothurn, Switzerland. The study was conducted in accordance with the Declaration of Helsinki and International Conference on Harmonization Guidelines in Good Clinical Practice and approved by the Ethics Committee of Northwest Switzerland (EKNZ), Swiss Federal Office for Public Health, and Swissmedic (Clinicaltrials.gov identifier: NCT03153579). The primary outcomes of this study have been previously published.^10^ In the present study, we report the long-term follow-up efficacy data and sustained effects of LSD in people with anxiety disorders.

Participants were recruited through an advertisement that was placed on website homepages of the University Hospital Basel and Swiss Medical Society for Psycholytic Therapy (SAePT) trial registries, or by word of mouth. All participants provided written informed consent before study inclusion. Written informed consent was obtained by the study psychiatrist who conducted the screening visit. The goal was to include people with anxiety disorders or significant anxiety that was associated with LTI. LTI was defined as any severe somatic disease, such as a diagnosis of cancer or another advanced-stage potentially fatal illness. Participants with LTI had to meet the *Diagnostic and Statistical Manual of Mental Disorders*, 4th edition (DSM-IV) criteria for an anxiety disorder, including generalised anxiety disorder, social phobia and panic disorder, as indicated by the Structured Clinical Interview for the DSM-IV (SCID-IV), or have a score ≥40 on the state or trait STAI at study inclusion. Participants without LTI had to meet DSM-IV criteria for at least one anxiety disorder. Thus, in people without LTI, elevated STAI scores were not sufficient for inclusion. All inclusion and exclusion criteria and participant characteristics have been previously reported^10^ and are described in the Supplementary Information.

After study inclusion, the participants were randomly assigned to LSD or placebo in the first treatment period and vice versa in the second treatment period by order of enrolment and group. LSD free base (>99% purity; Lipomed AG, Arlesheim, Switzerland) was administered as an oral solution in units that contained 100 μg LSD in 1 mL of 96% ethanol. Inactive placebo consisted of identical units that were filled with ethanol only. Randomisation and production were performed according to good manufacturing practice (GMP) by a licensed GMP facility (Apotheke Dr Hysek, Biel, Switzerland).

### Procedures

As previously reported in detail, the study included a screening visit and two 24-week treatment periods per participant. Each treatment period consisted of two treatment sessions and five study visits. Treatment sessions were separated by 6 weeks (±2 weeks). Study visits were conducted at baseline, between treatment sessions, and 2, 8 and 16 weeks after the second treatment session. The last visit in the second period also served as the end-of-study visit. Follow-up questionnaires were sent by mail to all participants (including dropouts) 12 months after the end-of-study visit.

Screening consisted of written informed consent, an evaluation of the individual's physical and mental health background, a psychiatric interview (SCID-IV), an assessment of anxiety severity, depression and further psychiatric symptomology, and a physical check-up. After successful screening, each person was assigned for the entire duration of the study to one investigator/therapist who conducted all treatment sessions and study visits. Treatment sessions lasted approximately 12 h, and study visits lasted approximately 1 h.

Study visits consisted of talk therapy, followed by an assessment of adverse events, changes in general medication and administration of the questionnaires.

Treatment sessions were conducted in a calm hospital room (University Hospital Basel) or calm practice room (Clinic Dr Peter Gasser). Only one participant and one investigator/therapist were present during the treatment sessions (exceptions of more than one therapist being present were made upon request by the therapist or participant). Details of the procedures have been previously published; see the Supplementary Information.^10^

The follow-up was conducted 12 months after the end-of-study visit by post to investigate the same outcome measures as during the study, in which participants completed questionnaires about long-lasting effects and answered questions about adverse events.

### Outcome measures

Outcome measures are described in detail in the Supplementary Information. The primary outcome measure in the present study was defined the STAI-G (with the primary end-point 16 weeks after the last treatment session, as reported previously).^10^ Secondary outcomes that were assessed during the study and at the follow-up were scores on the STAI-State (STAI-S), STAI-Trait (STAI-T), BDI and SCL-90-R. The clinical response was defined as a STAI-G reduction ≥ 30%. Further secondary end-points, which were reported in the primary analysis, were acute subjective drug effects during treatment sessions, assessed by the 5 Dimensional Altered States of Consciousness (5D-ASC) and Mystical Experience Questionnaire 30-item version (MEQ30).

At follow-up, in addition to the therapeutic outcome questionnaires, a questionnaire about persisting effects of psychedelics was administered. The 143-item Persisting Effects Questionnaire (PEQ) is a questionnaire that has previously been used to study positive and negative long-term effects of psilocybin and LSD.^15,22–25^ An earlier-published German version was used.^26^

Personality traits at screening and the 1-year follow-up were assessed using the 60-item NEO**-**Five-Factor Inventory (NEO-FFI)^27^ that was derived from the NEO Personality Inventory.^28^

Additionally, the follow-up questionnaire included questions about adverse effects, including flashbacks and hallucinogen persisting perception disorder (HPPD; see Supplemental Information for detailed questions), and participants were asked for further comments about the study.

### Data analyses

The sample size calculation was previously reported in detail.^10^ Data were analysed using R Studio software, version 2023.12.1+402. Outcomes were analysed using Linear Mixed Effects models, employing the lme4 and lmer test packages in R. Time points were used as the fixed factor, and participants were used as the random factor. For therapeutic outcomes but not personality traits, baseline values of the periods were considered covariates (change from baseline values for the respective period). Tukey's *post hoc* pairwise comparisons of least square means across time points were performed to elucidate significant differences between baseline and follow-up. These analyses were planned and hypothesised a priori in the protocol. Exploratory associations between the acute response to LSD and main follow-up outcome measures were assessed using Pearson's correlations. As similarly conducted in the previous primary analysis of this study.^10^ The criterion for significance was *P* < 0.05.

Qualitative data were summarised using an inductive approach and adding positive and negative valences.

## Results

Enrolment began on 23 June 2017 and finished on 1 February 2021. The trial ended as planned, with the last patient visit on 15 December 2021. The assessment of follow-up questionnaires was completed by January 2023. Detailed data on dropouts during the study were previously reported.^10^ On average, people were diagnosed with anxiety 10 ± 9 years (mean ± s.d.) prior to study inclusion (range: 0–39 years). Follow-up questionnaires were returned by 35 individuals (15 in the group with LTI and 20 without LTI). People who had at least two LSD sessions and one outcome measure were included in the analysis. One person did not complete the STAI questionnaires, resulting in a total of 33 people for all STAI measures and 34 people for all other measures. A total of 39 participants (20 in the LSD-first group and 19 in the placebo-first group) were eligible for the change-from-baseline analysis per treatment group. Six individuals from the original study continued psychedelic therapy (LSD, 3,4-methylenedioxymethamphetamine (MDMA) or psilocybin) within the Swiss limited-use programme and underwent one to four additional psychedelic sessions (all LSD).^26^ The long-term follow-up occurred 94 and 68 weeks after the last dose of LSD in the LSD-first and LSD-second groups, respectively.

**Fig. 1:**
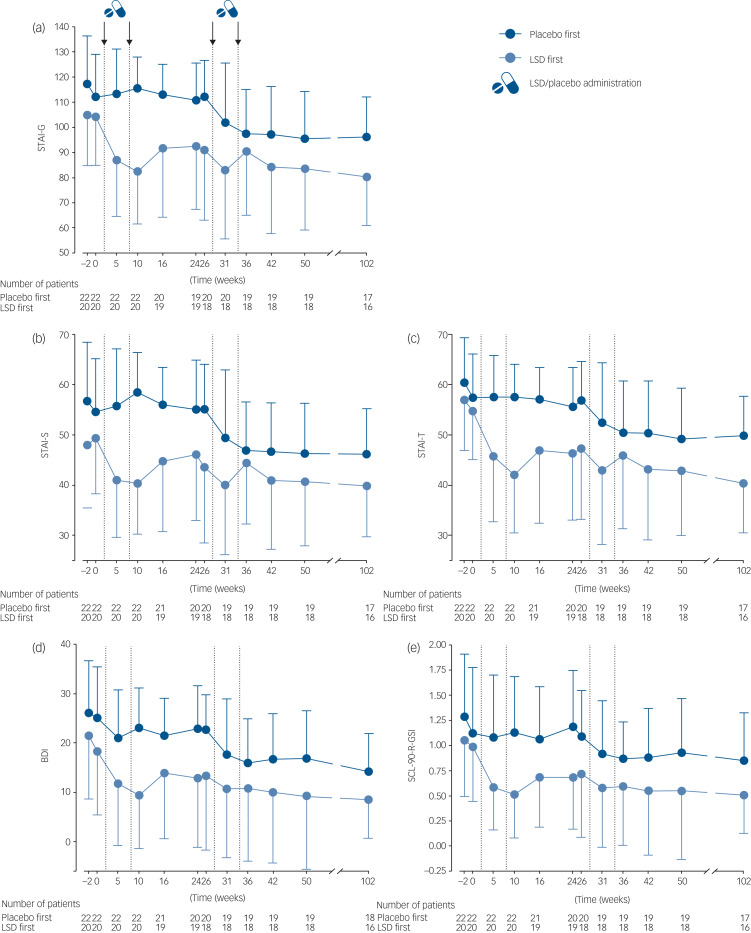
Outcome progress over the entire study duration, showing effects of lysergic acid diethylamide (LSD) and placebo on study outcome measures over time and during both treatment periods. In the LSD-first group, LSD produced strong effects that carried over into the second treatment period and were sustained up to week 102. In the placebo-first group, there were no relevant changes in scores in the first treatment period, and LSD was effective in the second treatment period with sustained effects. The total number of participants is shown in the graph. Screening occurred 2 weeks before the first baseline visit (week −2). Treatment sessions with either LSD (two sessions) or placebo (two sessions) occurred at weeks 2 and 8 in the first treatment period, and at weeks 28 and 34 in the second treatment period. The treatment crossover occurred after week 24. Outcome measures were assessed between sessions (weeks 5 and 31), and 2 weeks (weeks 10 and 36), 8 weeks (weeks 16 and 42) and 16 weeks (weeks 24 and 50) after the second treatment session per period. (a) Spielberger's State-Trait Anxiety Inventory–Global Score (STAI-G). (b) Spielberger's State-Trait Anxiety Inventory–State Score (STAI-S). (c) Spielberger's State-Trait Anxiety Inventory–Trait Score (STAI-T). (d) Beck Depression Inventory (BDI). (e) Symptom-Check-List-90-R (SCL-90-R). Values are absolute scores, expressed as means and standard deviations.

Participants reported sustained reductions of anxiety, depression and general psychiatric symptomatology up to 12 months after the end-of-study visit compared with baseline measures (Fig. 1, Table 1; individual courses are presented in Supplementary Figure 1, available at https://doi.org/10.1192/bjp.2024.99). The least square mean (95% CI) changes in the primary end-point measure (STAI-G score) from baseline to week 102 for the LSD-first group and from baseline to week 76 for the placebo-first group were −21.6 (−32.7, −10.4) and −16.5 (−26.2, −6.8), respectively, indicating significant reductions compared with baseline measures of the respective treatment group (*P* < 0.001 and *P* < 0.05 for the LSD-first and placebo-first groups, respectively). Effect sizes remained large in both groups (Cohens’ *d* = 1.04 and 1.02, respectively). The LSD-first group improved significantly on the STAI-G compared with the primary end-point (16 weeks after last drug administration) at follow-up. The least square mean (95% CI) change was −9.7 (−15.5, −4.0), while the placebo-first group showed no further improvement: 1.2 (−4.2, 6.6). *Post hoc* tests revealed no significant differences between people with and without LTI (*P* = 0.45 for the LSD-first group and *P* = 0.41 for the placebo-first group), people whose therapy was continued within the Swiss limited-use programme (*P* = 0.89 for the LSD-first group and *P* = 0.66 for the placebo-first group) and people with a formal diagnosis of generalised anxiety disorder (GAD; *P* = 0.35 for the LSD-first group and *P* = 0.65 for the placebo-first group).

**Table 1:** Outcomes as the change from baseline^a^

	STAI-G			STAI-S			STAI-T			BDI			SCL-90-R-GSI		
Weeks from baseline of treatment period	Difference from baseline of treatment period (95% CI)	*d*	*P*-value	Difference from baseline of treatment period (95% CI)	*D*	*P*-value	Difference from baseline of treatment period (95% CI)	*d*	*P*-value	Difference from baseline of treatment period (95% CI)	*d*	*P*-value	Difference from baseline of treatment period (95% CI)	*d*	*P*-value
LSD first group (*n* = 20)															
2 (LSD Session 1)															
5	−17.3 (−28.1, −6.4)	0.83		−8.3 (−13.9, −2.6)	0.73		−9.0 (−14.8, −3.2)	0.78		−6.5 (−11.3, −1.7)	0.51		−0.4 (−0.6, −0.2)	0.81	
8 (LSD Session 2)															
10	−21.7 (−32.5, −10.8)	1.08		−9.0 (−14.6, −3.3)	0.85		−12.7 (−18.5, −6.9)	1.18		−8.9 (−13.7, −4.1)	0.74		−0.5 (−0.7, −0.2)	0.94	
16	−13.2 (−24.1, −2.2)	0.47		−4.8 (−10.6, 0.9)	0.32		−8.3 (−14.2, −2.4)	0.58		−4.4 (−9.3, 0.5)	0.28		−0.3 (−0.5, −0.1)	0.52	
24	−12.4 (−23.3, −1.5)	0.47		−3.5 (−9.3, 2.2)	0.23		−8.9 (−14.8, −3.0)	0.67		−5.4 (−10.3, −0.6)	0.35		−0.3 (−0.5, −0.1)	0.52	
26	−13.4 (−24.4, −2.4)	0.47		−5.6 (−11.4, 0.1)	0.35		−7.8 (−13.7, −1.8)	0.55		−5.1 (−10.0, −0.1)	0.30		−0.3 (−0.5, 0.0)	0.39	
31	−21.3 (−32.3, −10.3)	0.81		−9.2 (−15, −3.4)	0.66		−12.1 (−18, −6.2)	0.88		−7.8 (−12.7, −2.9)	0.52		−0.4 (−0.6, −0.2)	0.64	
36	−14.0 (−25, −3.0)	0.53		−4.8 (−10.6, 1.0)	0.34		−9.2 (−15.1, −3.2)	0.64		−7.6 (−12.6, −2.7)	0.49		−0.4 (−0.6, −0.2)	0.63	
42	−20.3 (−31.3, −9.3)	0.78		−8.3 (−14, −2.5)	0.59		−12.0 (−17.9, −6.1)	0.90		−8.5 (−13.4, −3.5)	0.56		−0.4 (−0.7, −0.2)	0.66	
50	−20.8 (−31.8, −9.8)	0.85		−8.5 (−14.3, −2.7)	0.64		−12.3 (−18.2, −6.3)	0.98		−9.2 (−14.1, −4.3)	0.60		−0.4 (−0.7, −0.2)	0.62	
**102**	**−21.6** (**−32.7, −10.4)**	**1**.**04**	**<0.001**	**−8.4** (**−14.3, −2.5)**	**0**.**71**	**<0**.**05**	**−13.2** (**−19.2, −7.1)**	**1**.**33**	**<0**.**001**	**−8.1** (**−13.2, −3.1)**	**0**.**71**	**<0**.**01**	**−0.4** (**−0.6, −0.2)**	**0**.**88**	**<0**.**01**
Placebo first group (*n* = 19)															
2 (LSD Session 1)															
5	−10.8 (−20.2, −1.4)	0.51		−5.9 (−11.9, 0.1)	0.49		−4.9 (−9.0, −0.8)	0.44		−4.8 (−8.7, −0.9)	0.50		−0.2 (−0.4, 0)	0.37	
8 (LSD Session 2)															
10	−15.6 (−25.0, −6.2)	0.97		−8.2 (−14.2, −2.3)	0.87		−7.4 (−11.5, −3.2)	0.82		−7.3 (−11.2, −3.4)	0.90		−0.3 (−0.5, −0.1)	0.65	
16	−15.9 (−25.3, −6.5)	0.94		−8.4 (−14.4, −2.5)	0.89		−7.5 (−11.6, −3.3)	0.84		−6.5 (−10.5, −2.6)	0.80		−0.2 (−0.4, −0.1)	0.54	
24	−17.4 (−26.8, −8.0)	1.04		−8.8 (−14.7, −2.8)	0.92		−8.6 (−12.7, −4.5)	0.99		−6.4 (−10.3, −2.4)	0.74		−0.2 (−0.4, 0.0)	0.40	
**76**	**−16.5** (**−26.2, −6.8)**	**1**.**02**	**<0.05**	**−8.8** (**−14.9, −2.6)**	**0**.**89**	**<0**.**05**	**−7.7** (**−12.0, −3.4)**	**1**.**00**	**<0**.**05**	**−8.9** (**−12.9, −4.9)**	**1**.**21**	**<0**.**01**	**−0.3** (**−0.5, −0.1)**	**0**.**61**	**<0**.**05**

a. Values are score changes from baseline reported as least square mean (95% CI) of the respective treatment period. *P*-values are only provided for the a priori hypothesised outcomes.

*d*, effect size, Cohen's *d*; STAI-G, Spielberger's State-Trait Anxiety Inventory Global Score; STAI-S, Spielberger's State-Trait Anxiety Inventory State Score; STAI-T, Spielberger's State-Trait Anxiety Inventory Trait Score; BDI, Beck Depression Inventory; SCL–90-R-GSI, Symptom-Check-List-90-R Global Severity Score; LSD, lysergic acid diethylamide.

Rows shown in bold present the main outcome of this paper.

At follow-up, a total of 11 participants (33%) were in remission with regard to anxiety symptoms, and a total of 17 participants (49%) were in remission with regard to depression symptoms (Table 2), indicating potentially long-lasting and sustained effects of LSD.

**Table 2:** Response and remission rates over time

	STAI-G												BDI					
	Remission^a^						Response^b^						Remission^c^					
Weeks from first visit	LSD first		Placebo first		All		LSD first		Placebo first		All		LSD first		Placebo first		All	
	%	*n*	%	*n*	%	*n*	%	*n*	%	*n*	%	*n*	%	*n*	%	*n*	%	*n*
2 (LSD/placebo session 1)																		
5	30	6	5	1	17	7	35	7	0	0	17	7	50	8	9	2	26	10
8 (LSD/placebo session 2)																		
10	35	7	0	0	17	7	50	10	0	0	24	10	56	9	0	0	24	9
16	32	6	0	0	15	6	35	7	0	0	17	7	40	6	5	1	20	7
24	32	6	5	1	18	7	40	8	10	2	25	10	40	6	5	1	21	7
26	33	6	0	0	16	6	40	8	0	0	20	8	57	8	0	0	24	8
28 (placebo/LSD session 1)																		
31	50	9	20	4	34	13	50	10	25	5	38	15	64	9	25	5	41	14
34 (placebo/LSD session 2)																		
36	39	7	16	3	27	10	30	6	42	8	36	14	57	8	26	5	39	13
42	50	9	21	4	35	13	45	9	37	7	41	16	57	8	26	5	39	13
50	50	9	16	3	32	12	40	8	37	7	38	15	64	9	32	6	45	15
**102**	**56**	**9**	**12**	**2**	**33**	**11**	**56**	**9**	**35**	**6**	**45**	**15**	**56**	**9**	**42**	**8**	**49**	**17**

a. Remission was defined as score <80 for the STAI-G.

b. Response was defined as STAI-G score reduction of ≥30%.

c. Remission was defined as score <10 for the BDI; only patients with a score of ≥10 at screening were included. Percentages are based on the number of patients described in Fig. 1. Rows shown in bold present follow-up data.

STAI-G, Spielberger's State-Trait Anxiety Inventory Global Score; BDI, Beck Depression Inventory; LSD, lysergic acid diethylamide.

On the PEQ, high ratings of positive attitudes about life and/or self, positive mood changes, altruistic/positive social effects and positive behavioural changes were reported (all >47% of the total maximum score), whereas ratings of the negative counterparts remained low (all <10% of the total maximum score). Overall, the LSD-first group showed nominally higher ratings in positive items and nominally lower ratings in negative items compared with the placebo-first group (Table 3). On average, people rated the experience as ‘among the ten most personally meaningful experiences in life’, as ‘very much’ spiritually meaningful and as having positively influenced their sense of well-being or life satisfaction (Table 3).

**Table 3:** Long-term effects

	All						LSD first						Placebo first					
	Screening		Follow-up				Screening		Follow-up				Screening		Follow-up			
	mean	s.d.	mean	s.d.			mean	s.d.	mean	s.d.			mean	s.d.	mean	s.d.		
Persisting Effects Questionnaire^a^^,^ ^b^																		
Positive attitudes about life and/or self			57	24					62	24					53	23		
Positive mood changes			56	27					62	26					50	28		
Altruistic/positive social effects			47	26					53	27					42	25		
Positive behaviour changes			59	32					60	33					58	31		
Negative attitudes about life and/or self			3.6	8.7					2.3	6.5					4.7	11		
Negative mood changes			6.4	17					1.6	6.3					11	22		
Antisocial/negative social effects			8.7	10					9.1	8.8					8.4	11		
Negative behaviour changes			1.8	7.7					0.0	0.0					3.5	11		
How personally meaningful was the experience? (score range 1–8)			6.2	1.5					6.3	1.5					6.2	1.8		
How spiritually significant was the experience? (score range 1–6)			4.3	1.4					4.3	1.3					4.3	1.6		
Did the experience change your sense of well-being or life satisfaction? (score range − 3 to + 3, 0 = no change)			2.3	0.21					2.2	0.31					2.4	0.24		
NEO Five-Factor Inventory^c^					*P* value	df					*P* value	df					*P* value	df
Neuroticism (max score = 48)	32	6.3	26	7.8	**<0**.**0001**	35.1	30	5.8	23	7.3	**0**.**0019**	17.3	34	6.5	29	7.2	**0**.**0015**	17.3
Extraversion (max score = 48)	20	6.5	24	6.0	**0**.**002**	34.3	21	6.4	25	6.9	**0**.**0162**	14.7	20	6.8	23	5.1	0.0551	18.7
Openness (max score = 48)	37	5.7	37	4.3	0.918	31.4	36	5.5	37	4.4	0.958	13.3	37	5.9	37	4.3	0.989	18.2
Agreeableness (max score = 48)	32	5.5	32	5.8	0.663	33.6	33	4.9	35	5.2	0.865	14.9	31	6.0	30	5.7	0.799	17.6
Conscientiousness (max score = 48)	29	7.6	29	8.2	0.95	33.6	30	7.3	32	6.5	0.553	14.8	28	8.1	26	8.6	0.728	17.8

a. Data are expressed as percentage of maximum possible score.

b. *N* = 33.

c. Screening *N* = 39, Follow-up *N* = 33.

Rows shown in bold present significant values (*P* < 0.05).

LSD, lysergic acid diethylamide.

On the NEO-FFI, ratings of neuroticism significantly decreased at follow-up compared with study screening (*P* < 0.0001), and ratings of extraversion increased significantly (*P* = 0.002; Table 3).

Correlations between acute LSD effects and long-term outcomes showed a significant correlation between ΔSTAI-G measures and mystical-type experiences (total MEQ30 score; *r*_p_ = −0.342, *P* = 0.048) and an almost similar, but not significant, correlation with positive effects on the 5D-ASC dimension oceanic boundlessness (*r*_p_ = −0.332, *P* = 0.055), while negative effects on the 5D-ASC dimension anxious ego-dissolution showed no correlation (*r*_p_ = −0.244, *P* = 0.164). Additionally, positive measures of the PEQ (positive attitudes about life and/or self, positive mood changes and altruistic/positive social effects) also significantly correlated with mystical-type experiences (*r*_p_ = 0.420, *P* = 0.015, *r*_p_ = 0.454, *P* = 0.008, and *r*_p_ = 0.361, *P* = 0.039, respectively), and positive attitudes about life and/or self and positive mood changes also significantly correlated with positive acute effect ratings on the 5D-ASC dimension oceanic boundlessness (*r*_p_ = 0.368, *P* = 0.035, and *r*_p_ = 0.359, *P* = 0.040, respectively; Supplementary Table 1). Negative acute effect ratings on the 5D-ASC dimension anxious ego-dissolution remained neutral with regard to the PEQ outcomes (Supplementary Table 1).

No additional adverse events were reported in the follow-up period, and no additional clinically relevant flashback phenomena or HPPD occurred.

We received written feedback from 18 participants, which included the following topics: therapeutic potential of LSD (14 participants; 13 positive, one negative), setting and atmosphere (five participants; three positive, two negative), guide/therapist interaction (two participants; one positive, one negative), dose-related feedback (two participants; both negative (one participant reported the dose was too high, and one participant reported the dose was too low)), post-session effects (two participants; one positive, one negative) and comparison between dosing days (one participant; negative). Overall, the feedback was positive. Four participants were hoping for further experiences and 17 were grateful for their study participation. Predominantly negative feedback was given by two participants (one participant (dropout) reported an insufficient match with their therapist, and one participant (completed study) reported that the second treatment with LSD interrupted their constructive inner processes that were started with the first experience; this participant also reported that they did not like the setting at University Hospital Basel).

## Discussion

The present study revealed potential long-lasting benefits from LSD-assisted therapy in people with anxiety symptoms. More specifically, ratings of anxiety (reflected by STAI-G scores) showed sustained improvements 12 months after the end-of-study visit. Additionally, ratings of comorbid depression (BDI) and general psychiatric symptoms (SCL-90-R) remained low. People also attributed positive life changes to their LSD experience, and the personality trait neuroticism significantly decreased, whereas extraversion increased. This is the first modern study that reported long-term effects over a 12-month post-study period in individuals with a primary anxiety disorder who were treated with LSD. Most previous studies reported prospective follow-ups of no longer than 12 months or investigated psilocybin.^3,11,12^

The present findings did not confirm our hypotheses. We hypothesised that therapeutic effects would not last up to the 12-month follow-up. The findings are consistent with other studies that used psychedelics. One study reported long-term beneficial effects up to 4.5 years post-psilocybin administration on cancer-related psychiatric distress.^12^ The study used similar outcomes to the present study, such as STAI-S, STAI-T, BDI and PEQ, and reported similarly large effect sizes as reported herein. Remission rates for anxiety symptoms were higher but nearly identical for depression symptoms. However, remission rates for anxiety symptoms were calculated using different outcome measures. Additionally, the sample size was smaller (*n* = 15), and there were clear differences in the treated populations. For instance, the present study included not only participants with cancer-related distress but also those with other LTIs and a group of participants without any LTIs, which may lead to different treatment responses.^12^ Similarly, as reported in the present study, people attributed positive life changes to the psychedelic experience. This finding aligns with the high proportion of people in both trials who rated the psychedelic experience as among the most meaningful experiences in life and is consistent with studies in healthy volunteers, which also consistently demonstrated enduring positive effects.^15,23,25,29^ Healthy participants attributed positive life changes to their psychedelic experiences, mirroring findings from therapeutic studies.^15,25^ Notably, research in healthy participants reports shifts in personality traits, particularly an increase in the trait openness,^13,14^ but changes in agreeableness and conscientiousness have also been observed.^15,30^ In the present study, we observed changes in the personality traits neuroticism and extraversion but not in the other domains. Recent studies in participants with major depressive disorder who underwent psilocybin-assisted therapy reported decreases in neuroticism^16,17^ and introversion^16^ and increases in openness.^16^ Increases in openness have been the most consistent finding in patients and healthy participants after psychedelic administration.^13,14,16^ Interestingly, the individuals in the present study had very high values in the trait openness at study inclusion compared with reference values from the general population,^31^ which may have partly contributed to their initial interest in participating in the present study. From a therapeutic perspective, high neuroticism and low extraversion are associated with depression and anxiety disorders.^18,21^ Changes in these personality traits support the long-lasting therapeutic outcomes and indicate a modulatory deep-lying effect of LSD-assisted therapy.

The present study had two different treatment groups, one that received LSD in the first period and one that received LSD in the second period. However, it remains unclear why individuals in the LSD-first group appeared to benefit more from LSD-assisted therapy compared with those who received placebo first. The most obvious difference between the two groups was that the placebo-first group had higher ratings in symptoms at inclusion, and this coincidental difference remained throughout the entire trial. However, when looking at the change from baseline measures over the study duration, the difference became less pronounced and was unlikely to reach statistical significance. This also raises a question about treatment expectancy and whether people in the placebo-first group felt disadvantaged by not receiving LSD in the first period and whether this motive remained intact and influenced the subsequent therapeutic response. This should be addressed in future trials by adding established measures of expectancy such as the Credibility/Expectancy Questionnaire^32^ or the Stanford Expectations of Treatment Scale.^33^ Also, the two placebo sessions in the second period could have served as additional integration sessions in those who received LSD in the first period.

In the primary report of this trial,^10^ acute mystical-type experiences predicted the therapeutic outcome at the primary end-point (16 weeks post-LSD administration), and this is consistent with several previous studies in individuals with depression and anxiety, bringing the acute psychedelic experience to the centre of psychedelic-assisted therapy.^6,7,34^ Evidence of long-term prediction, however, is still unclear. Two recent long-term follow-ups in people with major depressive disorder or cancer-related psychological distress failed to show this relationship.^11,12^ In the present analysis, the correlations were not as strong as they were in the primary report,^10^ suggesting that other factors might influence the long-term therapeutic outcome. These factors could include aspects such as psychedelic-induced neuroplasticity, psychotherapeutic elements, environmental factors and other pharmacological or extra-pharmacological considerations which remain to be tested in future studies.

In the present study, six participants proceeded with psychedelic therapy (LSD, MDMA or psilocybin) within the Swiss limited-use programme after completion of the initial trial. There was no significant difference in the primary treatment outcome (STAI-G) at follow-up compared with those who had no further psychedelic-assisted therapy, although an important consideration is the small sample size (*n* = 6). This does not necessarily imply a lack of benefit from ongoing psychedelic-assisted therapy, but it might suggest that some individuals required additional sessions to maintain improved outcomes, but others did not.

LSD did not induce any long-term adverse events, such as flashbacks or HPPD. This aligns with several other studies and emphasises the general safety of LSD-assisted therapy.^3,10,26,35,36^

The present study included participants with a range of anxiety diagnoses that were included based on their symptom severity; this approach is supported by the findings that we found no difference in response between the groups (LTI versus non-LTI) and diagnosis (GAD versus non-GAD). The consistent efficacy across different anxiety diagnoses indicates the treatment's potential as a transdiagnostic intervention, suitable for a broad range of anxiety disorders without favouring any specific subgroup.

The present study has several strengths. We included individuals with anxiety symptoms with and without LTI and observed people over a relatively long study duration and a relatively long follow-up, having accumulated symptom data over 2 years for each participant. Furthermore, to date, this study is the largest psychedelic-assisted trial investigating long-term outcomes in a psychiatric population. The present study also has limitations. The present analysis included no control group for long-term effects of LSD. Additionally, no objective, interview-based data were collected, the herein-reported data are based on self-report questionnaires and no measure of expectancy was collected. All participants received LSD within this crossover study. Lasting effects were compared with measures before LSD-assisted therapy over time and within subjects. Therefore, we cannot exclude the possibility that other factors contributed to the long-term effects. Furthermore, it is largely unclear what therapy people received between the end-of-study visit and the follow-up. However, most people had already received some therapy (e.g. anxiolytics, antidepressants or talking psychotherapy) when they entered the trial, and antidepressant treatment was only tapered off for LSD/placebo sessions.^10^ LSD therapy therefore served as an add-on for those who had already received treatment at trial inclusion. Therefore, LSD-assisted therapy might have worked as a ‘door-opener’ to any kind of therapy and facilitated further therapy attempts. Many of the participants in the present trial had a long history of treatment attempts/failures and were to some degree non-responsive chronic cases.

In conclusion, 1 year after the last visit of a study that investigated LSD-assisted treatment in patients with anxiety disorders, symptoms of anxiety and depression remained low. Lasting negative effects were minimal. Patients reported enhanced well-being, and showed reduced neuroticism and increased extraversion.

## Supporting information

Holze et al. supplementary material Holze et al. supplementary material

## Data Availability

The data that support the findings of this study are available from the corresponding author, M.E.L., on reasonable request. Additional research material, i.e. to replicate Fig. 1, and analytic code are also available on reasonable quest.
