# Supplementary material for: LSD-assisted therapy in patients with anxiety: open-label prospective 12-month follow-up
Source: Br J Psychiatry. 2024 Sep;225(3):362–70. doi: 10.1192/bjp.2024.99 (PMC11536188; doi:10.1192/bjp.2024.99)
Supplement: Holze et al. supplementary material [file S0007125024000990sup001.docx]

**SUPPLEMENTAL INFORMATION**

**LSD-assisted therapy in patients with anxiety disorders: an open-label prospective 12-month follow-up**

**Supplemental Methods**

**Inclusion and exclusion criteria**

**Inclusion criteria**

1. Age > 25 years.

2. Meet DSM-IV criteria for anxiety disorder as indicated by the SCID-IV or have a score of at least 40 on the state- or trait STAI scale at study inclusion.

3. 40% or more of the participants should have a diagnosis of advanced-stage potentially fatal illness (autoimmune, neurological, or cancer without CNS involvement). Patients should be ambulatory and not terminal and likely to have a roughly estimated life expectancy of > twelve months.

4. Patients without advanced-stage potentially fatal illness need to meet DSM-IV criteria for anxiety disorder (elevated STAI score not sufficient for inclusion)

5. Sufficient understanding of the study procedures and risks associated with the study.

6. Participants must be willing to adhere to the study procedures and sign the consent form.

7. Participants are willing to refrain from taking any psychiatric medications during the experimental session period. If they are being treated with antidepressants or are taking anxiolytic medications on a fixed daily regimen such drugs must be discontinued long enough before the LSD/placebo treatment session to avoid the possibility of a drug-drug interaction (the interval will be at least 5 times the particular drug's half-life [typically 3-7 days]).

8. If in ongoing psychotherapy, those recruited into the study may continue to see their outside therapist, provided they sign a release for the investigators to communicate directly with their therapist. Participants should not change therapists, increase or decrease the frequency of therapy or commence any new type of therapy during the study (not including the follow-up).

9. Participants must also refrain from the use of any psychoactive drugs, with the exception of the long term pain medication or caffeine or nicotine, within 24 hours of each LSD/placebo treatment session. They must agree not to use nicotine for at least 2 hours before and 6 hours after each dose of LSD. They must agree to not ingest alcohol-containing beverages for at least 1 day before each LSD treatment session. Non-routine medications for treating breakthrough pain taken in the 24 hours before the LSD treatment session may result in rescheduling the treatment session to another date, with the decision at the discretion of the investigators after discussion with the participant.

10. Participants must be willing not to drive a traffic vehicle or to operate machines within 24 h after LSD/placebo administration.

**Exclusion criteria**

1. Women who are pregnant or nursing, or of child bearing potential and are not practicing an effective means of birth control (double-barrier method, i.e. pill/intrauterine device and preservative/diaphragm).

2. Past or present diagnosis of a primary psychotic disorder. Subjects with a first degree relative with psychotic disorders are also excluded.

3. Past or present bipolar disorder (DSM-IV)

4. Current substance use disorder (within the last 2 months, DSM-IV, except nicotine)

5. Somatic disorders including CNS involvement of the cancer, severe cardiovascular disease, untreated hypertension, severe liver disease (liver enzymes increase by more than 5 times the upper limit or normal) or severely impaired renal function (estimated creatinine clearance <30 ml/min), or other that in the judgement of the investigators pose too great potential for side effects

6. Weight < 45 kg

7. Suicide risk or likely to require psychiatric hospitalization during the course of the study

8. Requiring ongoing concomitant therapy with a psychotropic drug (other than as needed, anxiety medications, and pain control medications) and unable or unwilling to comply with the washout period.

**Procedures**

**Treatment Sessions**

Treatment sessions were conducted in a calm hospital room (University Hospital Basel) or in a calm practice room (Clinic Dr. Peter Gasser). Only one patient and one investigator/therapist were present during the treatment sessions (exceptions of more than one therapist being present were made upon request by the therapist or patient). The treatment sessions began at 8:00 AM. LSD or placebo was administered at 9:00 AM. The therapists and patients were in the same room for the whole treatment session, and a common meal was provided at approximately 6:00 PM. Afterward, acute subjective drug effects were assessed using the 5D-ASC and MEQ30 at the end of the session. At 8:00 PM, the patients were sent home in the company of a partner or friend. If necessary, then the patients could spend the night at the research facility, in which case an assistant was always on call who was ready to spend the night in the facility.

**Randomization and study drug**

After study inclusion, the patients were randomly assigned to LSD or placebo in the first treatment period and vice versa in the second treatment period by order of enrollment and group. Computerized block randomization (balanced blocks of 2 and 4 patients) was conducted by group (with or without a life threatening illness) and center and counter-balanced within group and center. LSD free base (> 99% purity; Lipomed AG, Arlesheim, Switzerland) was administered as an oral solution in units that contained 100 µg LSD in 1 ml of 96% ethanol^1^. Inactive placebo consisted of identical units that were filled with ethanol only. For allocation concealment medication was prepackaged and numbered by Patient-ID and Session (1-4). Randomization and production according to good manufacturing practice (GMP) were performed by a licensed GMP facility (Apotheke Dr. Hysek, Biel, Switzerland).

**Measures**

**Psychometric assessments**

*Spielberger’s State-Trait Anxiety Inventory (STAI)*

The STAI is a widely used and validated self-report instrument for assessing anxiety in adults. The STAI has been shown to be sensitive for the effects of LSD in a pilot study^2^ and sensitive for psilocybin in several previous studies ^3-6^. The STAI is available in many languages including a validated German version. It includes separate measures of state and trait anxiety.^7^ The STAI evaluates essential qualities of feelings of apprehension, tension, nervousness, and worry. The STAI differentiates between the temporary condition of state anxiety and the more general and long-standing quality of trait anxiety. The STAI state anxiety (STAI-S) subscale asks about feelings at the moment of completing the questionnaire. The STAI trait anxiety (STAI-T) subscale asks subjects to indicate how they generally view themselves. For both subscales, scores from 20 to 39 indicate mild anxiety, scores from 40 to 59 indicate moderate anxiety, and scores from 60 to 80 indicate severe anxiety. Both the STAI-S and STAI-T are commonly used as outcome measures in studies of patients with anxiety disorders.^8,9^ An STAI global (STAI-G) score can be derived by adding STAI-S and STAI-T scores (range: 40-160 points). The scoring in the present study was performed according to a previous study.^7^

*Hamilton Depression Rating Scale (HAM-D)*

The study psychiatrists assessed the patients’ depression severity using the 21-item HAM-D (HAM-D-21).^10,11^ This rating scale consists of 21 items (3- to 5-point ratings) that ask about symptoms related to depression, such as low mood, suicidality, irritability, tension, loss of appetite, insomnia, loss of interests, somatic symptoms, and similar. The summary scores were calculated as described previously.^10^

*Beck Depression Inventory (BDI)*

The BDI consists of 21 questions that were developed to measure the severity of depression.^12^ The German version of the BDI-II^13,14^ was used as a self-assessment. The BDI previously revealed an improvement of mood 6 months after psilocybin-assisted psychotherapy for anxiety in patients with advanced-stage cancer.^3^ Summary scores were calculated as described previously.^13^

*Symptom-Check-List-90-R (SCL-90-R)*

The SCL-90-R is a widely used psychological status symptom inventory^15,16^. We used the German version^16^. Outcome measures are the global severity index (SCL-90-R-GSI), positive symptom distress index (SCL-90-R-PSDI), and positive symptom total (SCL-90-R-PST). Reductions of these SCL-90-R scores were previously observed after LSD-assisted psychotherapy in patients with a life-threatening illness^2^. SCL-90 scores were calculated according to a previous study.^17^

**Subjective drug effect measurements**

Previous studies showed that positively experienced acute effects of psilocybin on the 5D-ASC and MEQ30 were associated with long-term therapeutic effects on anxiety and depression.^4,5,18^ Therefore, we hypothesized that acute effects of LSD on 5D-ASC Oceanic Boundlessness but not Anxious Ego-Dissolution^18^ and MEQ30 total scores would correlate with reductions of anxiety on the STAI-G scale 16 weeks after LSD.

*5 Dimensions of Altered States of Consciousness (5D-ASC) scale*

The 5D-ASC scale^19,20^ was administered once at the end of each treatment session to retrospectively rate peak drug effects. The 5D-ASC scale measures altered states of consciousness and contains 94 items that are assessed on visual analog scales. The instrument consists of five subscales/dimensions^19^ and 11 lower-order scales.^20^ The 5D-ASC Oceanic Boundlessness (OB) dimension (27 items) measures derealization and depersonalization that are associated with positive emotional states, ranging from heightened mood to euphoric exaltation. The corresponding lower-order scales include Experience of Unity, Spiritual Experience, Blissful State, Insightfulness, and Disembodiment. The Anxious Ego Dissolution (AED) dimension (21 items) summarizes ego disintegration and loss of self-control phenomena that are associated with anxiety. The corresponding lower-order scales include Impaired Control of Cognition and Anxiety. The Visionary Restructuralization (VR) dimension (18 items) consists of the lower-order scales Complex Imagery, Elementary Imagery, Audio-Visual Synesthesia, and Changed Meaning of Percepts. Two additional dimensions describe Auditory Alterations (AA; 15 items) and Reduction of Vigilance (VIR; 12 items). The total 3D-ASC (OAV) score is the total of the three main dimensions OB, AED, and VR and can be used as a measure of the overall intensity of alterations of mind.^21^ The scale is well-validated in German^19^ and many other languages and widely used to characterize subjective effects of various psychedelic drugs. The scale has been used by most research groups to psychometrically assess effects of LSD.^22-27^ Furthermore, acute positive effect ratings on the 5D-ASC (OB but not AED scores) after psilocybin administration have been used to predict long-term effects of psychedelic treatments in patients.^18^ Ratings on the 5D-ASC have been shown to closely correlate with ratings on the Mystical Effects Questionnaire (MEQ; see below),^21^ which is primarily used by research groups in the United States.^5^

*Mystical Effects Questionnaire*

Mystical experiences were assessed once at the end of the treatment sessions using the 100-item States of Consciousness Questionnaire (SOCQ)^21,28^ that includes the 30-item Mystical Effects Questionnaire (MEQ30).^29^ The published German version was used.^21^ The MEQ has been used in numerous experimental and therapeutic trials with psilocybin.^4,5,28,30-35^ The MEQ items provide scale scores for each of seven domains of mystical experiences: internal unity, external unity, sacredness, noetic quality (as real as or more real than everyday reality), deeply felt positive mood, transcendence of time and space, and ineffability/paradoxicality (difficulty describing the experience in words). The total of all scale scores was used as an overall measure of the mystical-type experience. We also derived the four scale scores of the newly validated revised 30-item MEQ: mystical, positive mood, transcendence of time and space, and ineffability^29^. Additionally, some aspects of the LSD experience may be better captured with this scale. For scale validation, see a previous study^29^. For the German translation of the MEQ30, see the online supplement in a previous study^21^.

*Persisting Effects Questionnaire*.

The 143-item PEQ is a non-validated questionnaire that has previously been used to study long-term effects of psilocybin and LSD ^28,31,32^. A previously published German version was used. A total of 140 items are rated on six-point Likert scales to assess possible changes in positive or negative attitudes about life and/or self (17 items each), positive and negative mood changes (4 items each), altruistic/positive and antisocial/negative social effects (8 items each), and positive and negative behavioral changes (1 item each; ^28^). All ratings reflect persisting changes that are subjectively related to the LSD experience, and no change would be reflected by a score of 0. Three additional questions were included as previously described ^28,31^: (1) How personally meaningful was the experience? (1 = no more than routine, everyday experiences; 2 = similar to meaningful experiences that occur on average once or more per week; 3 = similar to meaningful experiences that occur on average once per month; 4 = similar to meaningful experiences that occur on average once per year; 5 = similar to meaningful experiences that occur on average once every 5 years; 6 = among the 10 most meaningful experiences of my life; 7 = among the 5 most meaningful experiences of my life; 8 = the single most meaningful experience of my life), (2) Indicate the degree to which the experience was spiritually significant for you (1 = not at all; 2 = slightly; 3 = moderately; 4 = very much; 5 = among the 5 most spiritually significant experiences of my life; 6 = the single most spiritually significant experience of my life), and (3) Do you believe that the experience and your contemplation of the experience have led to a change in your current sense of personal well-being or life satisfaction? (+3 = increased very much to -3 = decreased very much; 0 indicates no change; ^28,31,32^.

*Assessment of Flashbacks and HPPD and Comments*

The questionnaire to assess flash-back phenomena and HPPD was conducted in German and consisted of the following questions (translated from German):

Did you experience flashbacks? (A flashback, in essence, translated as re-experiencing or echo memory, is a psychological phenomenon that can occur after an LSD experience. The affected person then has a sudden, usually powerful re-experiencing of events or emotional states as they occurred during the LSD experience. The term is used especially when the memory emerges involuntarily and/or when it is so intense that the person relives the experience.)

☐ YES ☐ NO

Did you experience persistent changes in perception? (Persistent changes in perception can occur after the consumption of consciousness-altering substances, such as altered spatial perception, seeing colors or patterns, etc.)

☐ YES ☐ NO

If you answered YES to flashbacks, please answer the following questions.

1. When did these flashbacks first occur in relation to the time duration after the last intake of LSD during the study?

_________________________________________________________________________________

2. How many flashbacks occurred (total number)? ____________________________

3. How long did each of these flashbacks last (seconds, minutes, hours)?

___________________________________________________________________________________________________________________________________________________________________________________________________________________________________________________

4. Over what period did the flashbacks occur (weeks, months, ongoing)?

___________________________________________________________________________________________________________________________________________________________________________________________________________________________________________________

5. Can you describe the flashbacks?

___________________________________________________________________________________________________________________________________________________________________________________________________________________________________________________

If you checked YES to persistent changes in perception, please answer the following questions

1. When did these changes in perception first occur in relation to the time duration after the last intake of LSD during the study?

___________________________________________________________________________________________________________________________________________________________________________________________________________________________________________________

2. Can you describe the changes in perception?

___________________________________________________________________________________________________________________________________________________________________________________________________________________________________________________

Do you have any further comments? :

_________________________________________________________________________________________________________________________________________________________

**Supplemental Results**


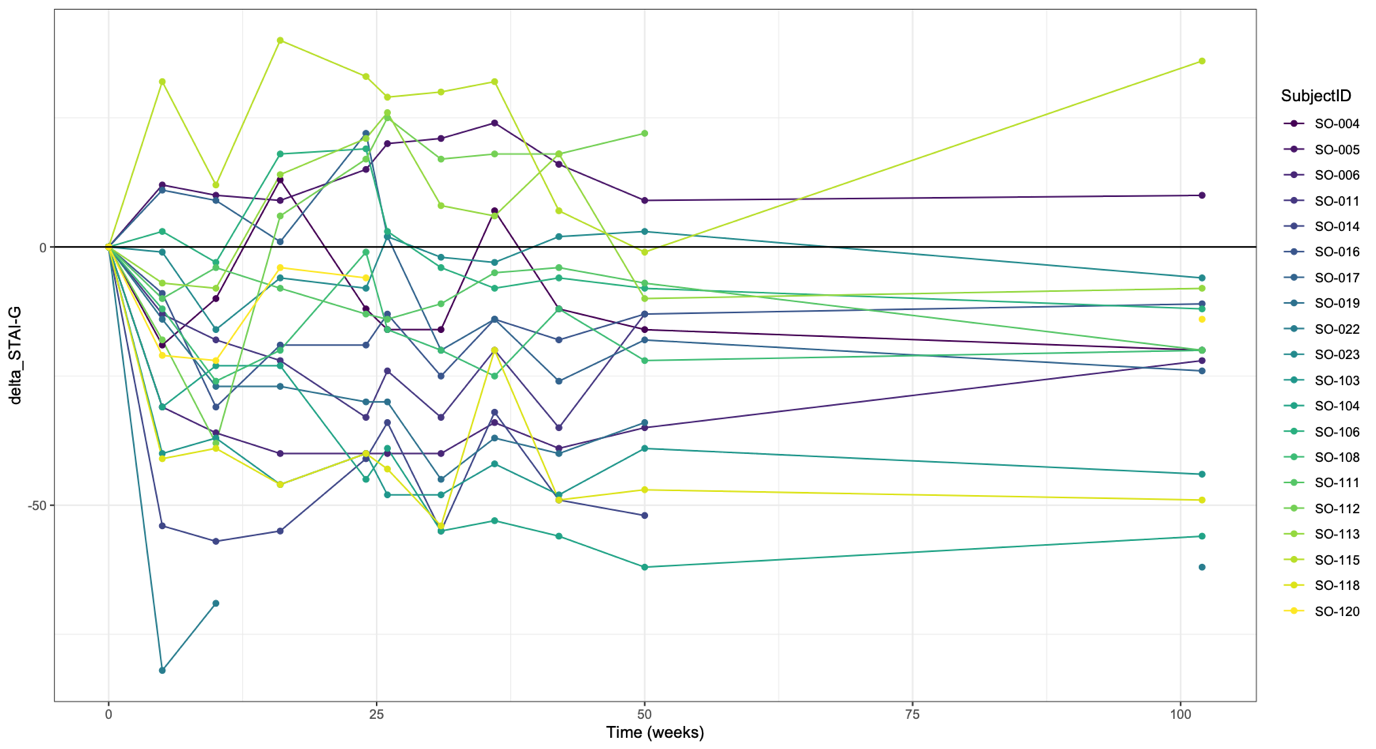
A


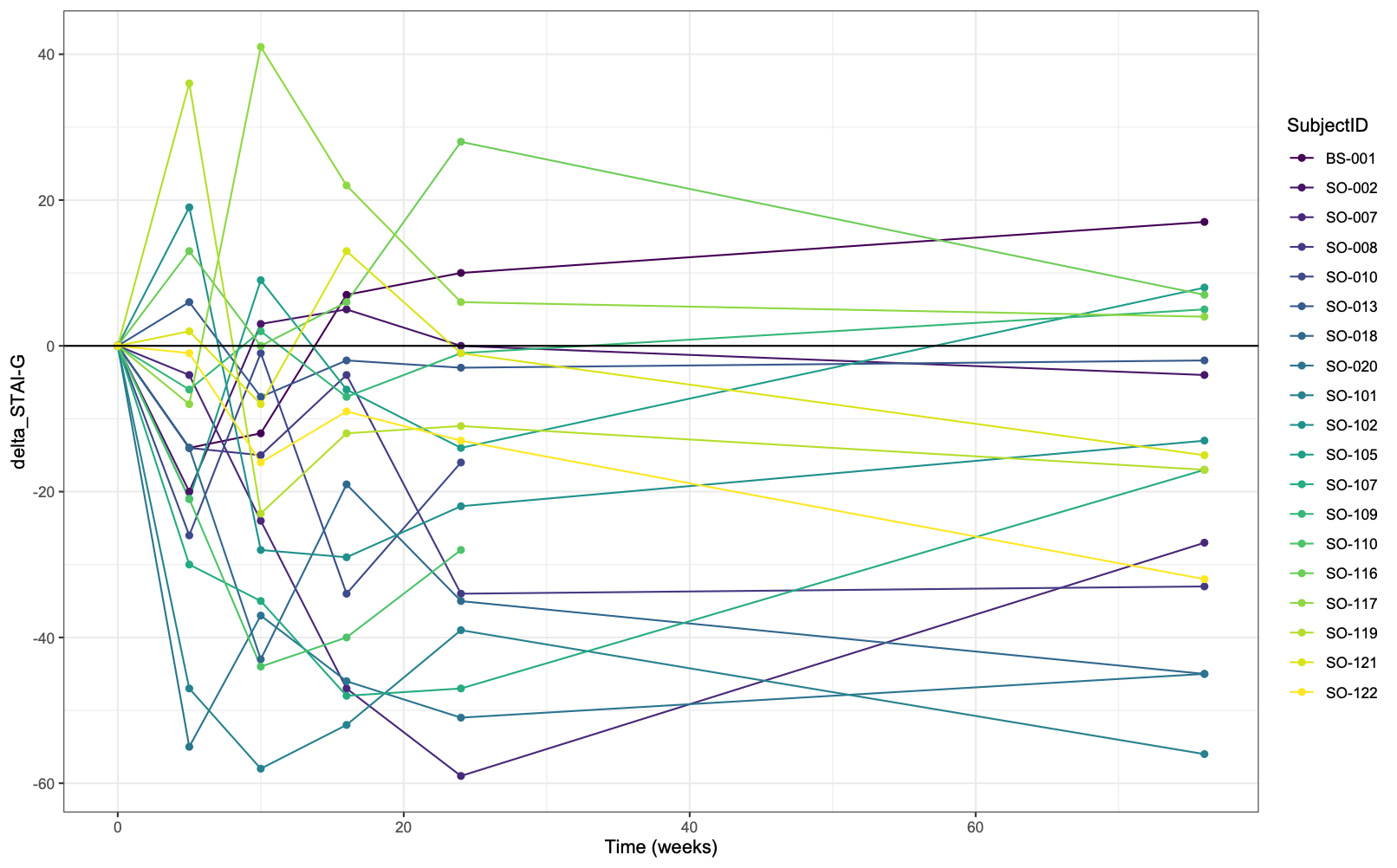
B

**Figure S1. Individual course**. Anxiety scores (STAI-G) for each of 39 study participants over time, as change from baseline of the respective treatment period. Individual participants are represented with different colors. **A** Patients who received LSD in the first treatment period. **B** Patients who received LSD in the second treatment period.

**References**

1. Holze F, Duthaler U, Vizeli P, Muller F, Borgwardt S, Liechti ME. Pharmacokinetics and subjective effects of a novel oral LSD formulation in healthy subjects. *British journal of clinical pharmacology* 2019; **85**: 1474-83.

2. Gasser P, Holstein D, Michel Y, Doblin R, Yazar-Klosinski B, Passie T, et al. Safety and efficacy of lysergic acid diethylamide-assisted psychotherapy for anxiety associated with life-threatening diseases. *The Journal of nervous and mental disease* 2014; **202**(7): 513-20.

3. Grob CS, Danforth AL, Chopra GS, Hagerty M, McKay CR, Halberstadt AL, et al. Pilot study of psilocybin treatment for anxiety in patients with advanced-stage cancer. *Archives of general psychiatry* 2011; **68**(1): 71-8.

4. Ross S, Bossis A, Guss J, Agin-Liebes G, Malone T, Cohen B, et al. Rapid and sustained symptom reduction following psilocybin treatment for anxiety and depression in patients with life-threatening cancer: a randomized controlled trial. *J Psychopharmacol* 2016; **30**(12): 1165-80.

5. Griffiths RR, Johnson MW, Carducci MA, Umbricht A, Richards WA, Richards BD, et al. Psilocybin produces substantial and sustained decreases in depression and anxiety in patients with life-threatening cancer: a randomized double-blind trial. *J Psychopharmacol* 2016; **30**(12): 1181-97.

6. Carhart-Harris R, Giribaldi B, Watts R, Baker-Jones M, Murphy-Beiner A, Murphy R, et al. Trial of psilocybin versus escitalopram for depression. *N Engl J Med* 2021; **384**(15): 1402-11.

7. Spielberger CD, Gorsuch RC, Lusheme RE. Manual for the Stait Trait Anxiety Inventory. Palo Alto: Consulting Psychologists Press; 1970.

8. Laakmann G, Schule C, Lorkowski G, Baghai T, Kuhn K, Ehrentraut S. Buspirone and lorazepam in the treatment of generalized anxiety disorder in outpatients. *Psychopharmacology (Berl)* 1998; **136**(4): 357-66.

9. Fisher PL, Durham RC. Recovery rates in generalized anxiety disorder following psychological therapy: an analysis of clinically significant change in the STAI-T across outcome studies since 1990. *Psychological medicine* 1999; **29**(6): 1425-34.

10. Hamilton M. A rating scale for depression. *Journal of neurology, neurosurgery, and psychiatry* 1960; **23**: 56-62.

11. Hamilton M. Rating depressive patients. *The Journal of clinical psychiatry* 1980; **41**(12 Pt 2): 21-4.

12. Beck AT, Ward CH, Mendelson M, Mock J, Erbaugh J. An inventory for measuring depression. *Archives of general psychiatry* 1961; **4**: 561-71.

13. Hautzinger M, Keller F, Kühner C. BDI II: Beck Depressions-Inventar: Revision. Frankfurt/Main, Germany: Harcourt Test Services; 2006.

14. Kuhner C, Burger C, Keller F, Hautzinger M. [Reliability and validity of the Revised Beck Depression Inventory (BDI-II). Results from German samples]. *Nervenarzt* 2007; **78**(6): 651-6.

15. Derogatis LR, Rickels K, Rock AF. The SCL-90 and the MMPI: a step in the validation of a new self-report scale. *Br J Psychiatry* 1976; **128**: 280-9.

16. Schmitz N, Hartkamp N, Kiuse J, Franke GH, Reister G, Tress W. The Symptom Check-List-90-R (SCL-90-R): a German validation study. *Qual Life Res* 2000; **9**(2): 185-93.

17. Franke GH. SCL-90-R: Symptom-Checkliste von L.R. Derogatis: Deutsche Version. Göttingen, Germany: Beltz Test GmbH; 2002.

18. Roseman L, Nutt DJ, Carhart-Harris RL. Quality of acute psychedelic experience predicts therapeutic efficacy of psilocybin for treatment-resistant depression. *Front Pharmacol* 2017; **8**: 974.

19. Dittrich A. The standardized psychometric assessment of altered states of consciousness (ASCs) in humans. *Pharmacopsychiatry* 1998; **31 (Suppl 2)**: 80-4.

20. Studerus E, Gamma A, Vollenweider FX. Psychometric evaluation of the altered states of consciousness rating scale (OAV). *PLoS One* 2010; **5**(8): e12412.

21. Liechti ME, Dolder PC, Schmid Y. Alterations in conciousness and mystical-type experiences after acute LSD in humans. *Psychopharmacology* 2017; **234**: 1499-510.

22. Carhart-Harris RL, Kaelen M, Bolstridge M, Williams TM, Williams LT, Underwood R, et al. The paradoxical psychological effects of lysergic acid diethylamide (LSD). *Psychological medicine* 2016; **46**: 1379-90.

23. Schmid Y, Enzler F, Gasser P, Grouzmann E, Preller KH, Vollenweider FX, et al. Acute effects of lysergic acid diethylamide in healthy subjects. *Biol Psychiatry* 2015; **78**(8): 544-53.

24. Dolder PC, Schmid Y, Mueller F, Borgwardt S, Liechti ME. LSD acutely impairs fear recognition and enhances emotional empathy and sociality. *Neuropsychopharmacology* 2016; **41**: 2638-46.

25. Holze F, Vizeli P, Muller F, Ley L, Duerig R, Varghese N, et al. Distinct acute effects of LSD, MDMA, and D-amphetamine in healthy subjects. *Neuropsychopharmacology* 2020; **45**(3): 462-71.

26. Bershad AK, Schepers ST, Bremmer MP, Lee R, de Wit H. Acute subjective and behavioral effects of microdoses of lysergic acid diethylamide in healthy human volunteers. *Biol Psychiatry* 2019; **86**(10): 792-800.

27. Preller KH, Herdener M, Pokorny T, Planzer A, Kraehenmann R, Stämpfli P, et al. The fabric of meaning and subjective effects in LSD-induced states depend on serotonin 2A receptor activation *Curr Biol* 2017; **27**: 451-57.

28. Griffiths RR, Richards WA, McCann U, Jesse R. Psilocybin can occasion mystical-type experiences having substantial and sustained personal meaning and spiritual significance. *Psychopharmacology (Berl)* 2006; **187**(3): 268-83; discussion 84-92.

29. Barrett FS, Johnson MW, Griffiths RR. Validation of the revised Mystical Experience Questionnaire in experimental sessions with psilocybin. *J Psychopharmacol* 2015; **29**(11): 1182-90.

30. MacLean KA, Johnson MW, Griffiths RR. Mystical experiences occasioned by the hallucinogen psilocybin lead to increases in the personality domain of openness. *J Psychopharmacol* 2011; **25**(11): 1453-61.

31. Griffiths RR, Johnson MW, Richards WA, Richards BD, McCann U, Jesse R. Psilocybin occasioned mystical-type experiences: immediate and persisting dose-related effects. *Psychopharmacology* 2011; **218**(4): 649-65.

32. Griffiths R, Richards W, Johnson M, McCann U, Jesse R. Mystical-type experiences occasioned by psilocybin mediate the attribution of personal meaning and spiritual significance 14 months later. *J Psychopharmacol* 2008; **22**(6): 621-32.

33. Garcia-Romeu A, Griffiths RR, Johnson MW. Psilocybin-occasioned mystical experiences in the treatment of tobacco addiction. *Current drug abuse reviews* 2014; **7**(3): 157-64.

34. Garcia-Romeu A, Davis AK, Erowid F, Erowid E, Griffiths RR, Johnson MW. Cessation and reduction in alcohol consumption and misuse after psychedelic use. *J Psychopharmacol* 2019; **33**(9): 1088-101.

35. Griffiths RR, Johnson MW, Richards WA, Richards BD, Jesse R, MacLean KA, et al. Psilocybin-occasioned mystical-type experience in combination with meditation and other spiritual practices produces enduring positive changes in psychological functioning and in trait measures of prosocial attitudes and behaviors. *J Psychopharmacol* 2018; **32**: 49-69.
